# Correction: Transcriptomics analysis of the bovine endometrium during the perioestrus period

**DOI:** 10.1371/journal.pone.0324686

**Published:** 2025-05-20

**Authors:** Mohammed A. Alfattah, Carolina N. Correia, John A. Browne, Paul A. McGettigan, Katarzyna Pluta, Stephen D. Carrington, David E. MacHugh, Jane A. Irwin

After the publication of this article [1], concerns were raised about identical tables across distinct time points and contradictory statements. Here, the authors have provided additional information to clarify these issues:

In the Results section of this paper, Tables 1 and 2 contain duplicate data as that of Table 4 by error. Tables 1–5 -5 are intended as summaries of the data presented in S3 Table, which are correct. Please see the corrected Tables 1 and 2 here.

**Table 1:** Top DEGs in bovine endometrium at 12 h post-CIDR removal versus oestrus, ranked by ascending B-H FDR.

Entrez ID	Gene Name	Gene Symbol	Log_2_ fold-change	*P*-Value	B-H FDR
**Upregulated**					
617325	kinase suppressor of ras 2	*KSR2*	4.71	1.13 × 10^−13^	4.80 × 10^−10^
282248	hypocretin (orexin) receptor 1	*HCRTR1*	3.23	2.00 × 10^−12^	2.60 × 10^−09^
509642	tubulointerstitial nephritis antigen-like 1	*TINAGL1*	2.14	4.63 × 10^−09^	2.53 × 10^−06^
522479	ovalbumin	*LOC522479*	4.54	6.04 × 10^−09^	3.19 × 10^−06^
282361	solute carrier family 5 (sodium/glucose cotransporter), member 1	*SLC5A1*	5.91	8.10 × 10^−09^	4.15 × 10^−06^
615277	acyl-coenzyme A thioesterase THEM4	*LOC615277*	6.26	9.06 × 10^−09^	4.38 × 10^−06^
104974435	uncharacterized LOC104974435	*LOC104974435*	6.38	1.16 × 10^−08^	5.02 × 10^−06^
786974	beta-hexosaminidase subunit beta	*LOC786974*	2.79	1.94 × 10^−08^	7.82 × 10^−06^
613555	disrupted in schizophrenia 1	*DISC1*	2.00	3.05 × 10^−08^	1.17 × 10^−05^
101905783	uncharacterized LOC101905783	*LOC101905783*	4.34	5.19 × 10^−08^	1.76 × 10^−05^
**Downregulated**					
509600	glycoprotein (transmembrane) nmb	*GPNMB*	−3.92	4.61 × 10^−17^	7.81 × 10^−13^
280852	midkine (neurite growth-promoting factor 2)	*MDK*	−2.13	3.97 × 10^−16^	3.36 × 10^−12^
353114	neuronatin	*NNAT*	−3.22	1.10 × 10^−13^	4.80 × 10^−10^
519120	pyridine nucleotide-disulphide oxidoreductase domain 2	*PYROXD2*	−2.2	2.98 × 10^−13^	1.01 × 10^−09^
531699	aldehyde oxidase 2	*AOX2*	−5.85	3.90 × 10^−13^	1.10 × 10^−09^
513038	cathepsin K	*CTSK*	−2.81	7.59 × 10^−13^	1.61 × 10^−09^
616777	family with sequence similarity 180, member A	*FAM180A*	−3.87	6.75 × 10^−13^	1.61 × 10^−09^
511839	ATPase, H+ transporting, lysosomal 38kDa, V0 subunit d2	*ATP6V0D2*	−5.69	8.94 × 10^−13^	1.68 × 10^−09^
789851	histamine receptor H3	*HRH3*	−6.41	1.21 × 10^−12^	2.04 × 10^−09^
100140069	WD repeat domain 86	*WDR86*	−3.11	1.68 × 10^−12^	2.54 × 10^−09^

**Table 2:** Top DEGs in bovine endometrium at 24 h post-CIDR removal versus oestrus, ranked by ascending B-H FDR.

Entrez ID	Gene Name	Gene Symbol	Log_2_ fold-change	*P*-Value	B-H FDR
**Upregulated**					
617325	kinase suppressor of ras 2	*KSR2*	3.42	7.62 × 10^−09^	1.07 × 10^−05^
615152	chromosome 14 open reading frame, human C8orf34	*C14H8orf34*	3.0	8.62 × 10^−07^	4.42 × 10^−04^
101906467	uncharacterized LOC101906467	*LOC101906467*	3.22	1.29 × 10^−06^	6.22 × 10^−04^
506696	paraspeckle component 1	*PSPC1*	0.95	1.46 × 10^−06^	6.84 × 10^−04^
280894	placental growth factor	*PGF*	1.81	1.63 × 10^−06^	7.28 × 10^−04^
530102	collagen, type VI, alpha 6	*COL6A6*	7.16	2.08 × 10^−06^	9.03 × 10^−04^
282361	solute carrier family 5 (sodium/glucose cotransporter), member 1	*SLC5A1*	4.48	2.85 × 10^−06^	1.15 × 10^−03^
616625	aquaporin 12B	*AQP12B*	5.12	6.13 × 10^−06^	2.21 × 10^−03^
286767	parathyroid hormone-like hormone	*PTHLH*	1.96	8.81 × 10^−06^	2.98 × 10^−03^
530709	Usher syndrome 1C (autosomal recessive, severe)	*USH1C*	2.96	9.39 × 10^−06^	3.07 × 10^−03^
**Downregulated**					
353114	neuronatin	*NNAT*	−3.19	1.92 × 10^−13^	3.25 × 10^−09^
509600	glycoprotein (transmembrane) nmb	*GPNMB*	−3.28	7.72 × 10^−13^	6.53 × 10^−09^
789851	histamine receptor H3	*HRH3*	−6.35	1.84 × 10^−12^	1.04 × 10^−08^
616010	anthrax toxin receptor 1	*ANTXR1*	−2.15	3.25 × 10^−12^	1.37 × 10^−08^
519120	pyridine nucleotide-disulphide oxidoreductase domain 2	*PYROXD2*	−1.95	9.78 × 10^−11^	3.31 × 10^−07^
281487	premelanosome protein	*PMEL*	−3.34	2.09 × 10^−10^	5.88 × 10^−07^
522392	matrix-remodelling associated 8	*MXRA8*	−2.12	9.91 × 10^−10^	2.39 × 10^−06^
508098	uncharacterized LOC508098	*LOC508098*	−7.26	1.98 × 10^−09^	4.19 × 10^−06^
338446	peroxisome proliferator-activated receptor gamma, coactivator 1 alpha	*PPARGC1A*	−2.73	2.80 × 10^−09^	5.26 × 10^−06^
616660	tetraspanin 11	*TSPAN11*	−1.79	3.15 × 10^−09^	5.33 × 10^−06^

The authors have provided the following corrections

In the Discussion section, the fourth paragraph under the subheading “Tissue remodelling and innervation in the proliferative phase” is incorrect. This paragraph should be disregarded.

In the Discussion section, the statement comprising the fourth and fifth sentences in the seventh paragraph under the subheading “Tissue remodelling and innervation in the proliferative phase” is incorrect. These sentences should be disregarded. The corrected paragraph is:

“For both time points before oestrus, transforming growth factor beta 1 (TGF-β1), the cytokine tumour necrosis factor (TNF), and HDAC (group of histone deacetylases) were among the top ranked upstream regulators (Table 7). TGF-β1 is required for the development of smooth muscle in the female reproductive tract [93], as well as being present in seminal fluid and altering the endometrium and oviduct to promote embryonic development and implantation [94]. In humans, expression of TNF has also been shown to rise in the mid- to late-proliferative phases [95], while in bovine endometrial cells, TNF-induced production of chemokines [96], directs the migration of leukocytes to inflammation sites [97]. These observations suggest that endometrial immunity is upregulated at this timepoint, actively preparing for sperm deposition and pathogen entry. The top interaction networks for CIDR+12 h and CIDR+24 h are centred on the transcription factor NFκB complex (Figs 4 and 5) although these are different networks. For the CIDR+12 h top network, involved in Endocrine system disorders, Hereditary disorders and Organismal injury and abnormalities, NF-κB is linked to various hub genes, such as *SUSD4*, which negatively regulates complement activation, and *ALDH1B1*, which links to various aldehyde dehydrogenases. The top network for CIDR+24 h is Neurological disease, Nervous System Development and Function and Behavior, with links to voltage-dependent Na+ channels and SYN genes, encoding synapsins that are associated with the trafficking of synaptic vesicles.”
