# Supplementary material for: Transcriptomics analysis of the bovine endometrium during the perioestrus period
Source: PLoS One. 2024 Mar 28;19(3):e0301005. doi: 10.1371/journal.pone.0301005 (PMC10977793; doi:10.1371/journal.pone.0301005)
Supplement: Attachment — Submitted filename: Response to Reviewers 2.docx [file pone.0301005.s008.docx]

**Response to Reviewers**

**Responses to Editor’s Comments (Comments are in blue, responses are in black text)**

1. When submitting your revision, we need you to address these additional requirements. Please ensure that your manuscript meets PLOS ONE's style requirements, including those for file naming.

We have revised our manuscript style in line with these, in particular with respect to headings, font size and file naming.

2. We note that the grant information you provided in the ‘Funding Information’ and ‘Financial Disclosure’ sections do not match.

When you resubmit, please ensure that you provide the correct grant numbers for the awards you received for your study in the ‘Funding Information’ section.

We have checked the grant numbers and provided the same grant data in the Funding Information and Financial Disclosure sections.

The Financial Disclosure text now reads as follows:

This work was supported by a grant to MAA from the Saudi Arabian Cultural Bureau (No. IR12012/2 and IR1610; funder website <https://ie.moe.gov.sa/ar/Pages/default.aspx> ) and a Brazilian Science Without Borders – CAPES grant (No: BEX-13070-13-4; funder website <https://www.gov.br/cnpq/pt-br/acesso-a-informacao/acoes-e-programas/programas/ciencia-sem-fronteiras> ) to CNC. The funders had no role in study design, data collection and analysis, decision to publish, or preparation of the manuscript.

3. **Data availability, repository information**

In your Data Availability statement, you have not specified where the minimal data set underlying the results described in your manuscript can be found.

4. We note that you have stated that you will provide repository information for your data at acceptance. Should your manuscript be accepted for publication, we will hold it until you provide the relevant accession numbers or DOIs necessary to access your data. If you wish to make changes to your Data Availability statement, please describe these changes in your cover letter and we will update your Data Availability statement to reflect the information you provide.

Our datasets are deposited in two locations: firstly, the raw RNA-seq data are deposited in the European Nucleotide Archive (ENA) and secondly, the bioinformatics and statistical workflow scripts are available from a public GitHub repository.

These datasets comprise the minimal dataset underlying our work. We have now made the ENA data public.

The ENA data are deposited under accession number PRJEB33671.

The following text is provided at <https://github.com/carolcorreia/Estrus-Endometrium-RNA-sequencing> :

**Oestrus-Endometrium-RNA-sequencing**

Repository containing all custom pipeline, perl scripts and R scripts used for RNA-sequencing data analyses of the Oestrus Endometrium project.

These will be published as part of a submitted publication: Alfattah, M. et al. (writing process)

In order to use the provided pipeline and scripts, user should refer first to the RNA-seq_pipeline_mohammed.txt file, which will lead to the other scripts. Further README files may be added for each scripts at later date, in order to facilitate user's utilisation and understanding.

For any inquirements concerning our article or repository, please contact:

Correia, C.N. carolina.correia [at] ucdconnect [dot] ie or MacHugh, D.E. david.machugh [at] ucd [dot] ie

**We have amended our Data Availability statement to read as follows:**

All of the bioinformatics and statistical workflow scripts (Bash,
Perl, and R programming languages) used are available from a public
GitHub repository <https://github.com/carolcorreia/Estrus-Endometrium-RNA-sequencing>

All RNA-Seq raw data used in this paper are available from the European Nucleotide Archive (ENA, <https://www.ebi.ac.uk/ena/browser/search> ) with accession number PRJEB33671

**Copyrighted images.** We note that Figure(s) 4, 5, 6, 7 and 8 in your submission contain copyrighted images. All PLOS content is published under the Creative Commons Attribution License (CC BY 4.0), which means that the manuscript, images, and Supporting Information files will be freely available online, and any third party is permitted to access, download, copy, distribute, and use these materials in any way, even commercially, with proper attribution. For more information, see our copyright guidelines: <http://journals.plos.org/plosone/s/licenses-and-copyright>. We require you to either (1) present written permission from the copyright holder to publish these figures specifically under the CC BY 4.0 license, or (2) remove the figures from your submission:

You may seek permission from the original copyright holder of Figure(s) 4, 5, 6, 7 and 8 to publish the content specifically under the CC BY 4.0 license.

We have requested information from the copyright holder, TS Bioinformatics at Qiagen. This has been granted and we have received a letter from the copyright holder with permission to publish the IPA network images (Figs 4-8). This has been uploaded as part of our submission as an ‘Other’ file. The figure legends have also been amended as requested, incorporating the following text: ‘Reprinted from Qiagen IPA under a CC BY license, with permission from Qiagen, original copyright 2017’.

5. **Image resolution**

We have improved the resolution of the images and have made some changes to Fig. 3 to improve legibility. The underlying graphs are the same (minus the axis labels) but were increased in size using the Photoshop image size feature with Smart Sharpen. Illustrator was then used to add editable text for the axis scales, labels, and key. The Fig 3. legend has also been expanded to make it clearer for the reader, as Reviewer 2 had concerns about the clarity of the legends.

**Responses to Reviewers’ Questions:**

**Reviewer 1:**

‘Did you only sample intercaruncular tissue or both caruncular and intercaruncular tissue?’.

Both tissue types were sampled, due to a difficulty in differentiating between these at the time. To clarify this for the reader, the following line has been inserted (lines 144-145 in final version): Both caruncular and intercaruncular tissues were sampled.

**Reviewer 2:**

Major Comments
The reviewer basically understands that this study is based on endometrial samples collected in the previous report (Pluta et. al, 2012, Physiol Genomics). However, the description of this manuscript does not make it clear to what extent the data are from the past and to what extent they were conducted in this study.
Pluta et. al (2012, Physiol Genomics) also performs RNA-seq. Are you using their RNA-seq data for IPA analysis?　Or are RNA extraction, library preparation and RNA-seq also performed in this study?　These should be clearly stated.

The samples from the reproductive tracts of the animals were taken from the cervix and endometrium on the same date. The endometrial samples were banked and frozen at -80°C for use as part of a later project, which formed part of Mohammed Alfattah’s Ph.D. This was done separately from the work described by Pluta et al. (2012) for cervical samples. The RNA extraction from the frozen endometrial samples was done later by MAA. Library preparation, RNA-seq and bioinformatic analysis were also done by Pluta and colleagues before MAA commenced his project. Therefore, the RNA-seq data, and their analysis for this manuscript were obtained as part of a separate project and compared to the data of Pluta et al. (2012) in the Discussion section of this manuscript. The text on lines 149-150 in the final version has been amended to explain this.

Figures and tables should be grouped together at the end of the manuscript. In the current version of the manuscript, reviewers were unable to understand the main text or legend. Are L276-278, L376-377 and L384-388 main text in Results section? It is very difficult to read.

The figure legends were embedded with the text, as this format is requested in the manuscript layout instructions for PLOS One. We agree that it can cause difficulties in interpreting the details of figures when a figure and its corresponding legend are not supplied in close proximity for easier reading.

Please provide a more detailed explanation of Figure legends for the benefit of the readers. In the current version of legends, it takes time to understand the figures and tables.

Amendments have been made to all figure legends, particularly that for Fig. 3, which has been extended significantly to provide a better explanation of what each panel in the figure means. Titles and descriptions for Tables have also been altered to provide more information for the reader. The changes made can be viewed on the marked-up copy of the manuscript.


**Minor Comments**
L137: …according to the methods of Cooke et. al (43) and Forde et. al (20).

This has been amended as advised.

L246-247: What is differences in “multiple locations” and “many locations” ?

‘Multiple locations’ refers to several; ‘too many locations’ refers to multi-mapping to 10 or more locations. This has now been more clearly defined in the text.

L262-268: The number of increased and decreased genes listed in parentheses should be deleted, because there were listed already in Figure 1A.

These have been deleted.

L364, 370, 378, 390 and 397: Are “Network 1” in each sections meaning the rank 1 network analyzed by IPA in each comparison? If so, should be described more clearly in the Figure legends.

This is correct. See amended figure legend for details.

L405: HDAC

This has been changed on this line, and also where it appears elsewhere, from Hdac to HDAC.

L407: Remove space before “S7”.

This has been done.

L445: (40, 68)

This has been changed to ‘by Pluta et al. [40] and Pluta [68]’, now on line 473 in manuscript.

L461: …by Bauersachs et. al (64) and Mitko et. al (3) ...

This has been changed as requested.

L566: HDAC See above.

L953-954: Ref. 68 Is this journal, book or thesis?

This reference refers to Kasia Pluta’s UCD Ph.D thesis (2011) and this detail has now been added to the Reference list.
